# Cancer-Associated Fibroblasts in a Human HEp-2 Established Laryngeal Xenografted Tumor Are Not Derived from Cancer Cells through Epithelial-Mesenchymal Transition, Phenotypically Activated but Karyotypically Normal

**DOI:** 10.1371/journal.pone.0117405

**Published:** 2015-02-06

**Authors:** Mei Wang, Chun-Ping Wu, Jun-Yan Pan, Wen-Wei Zheng, Xiao-Juan Cao, Guo-Kang Fan

**Affiliations:** 1 Department of Otolaryngology, Shanghai Municipal Hospital of Traditional Chinese Medicine, Shanghai, People’s Republic of China; 2 Department of Otolaryngology, the Second Affiliated Hospital of Jiaxing University, Jiaxing, Zhejiang Province, People’s Republic of China; 3 Department of Otolaryngology-Head and Neck Surgery, Eye, Ear, Nose and Throat Hospital of Fudan University, Shanghai, People’s Republic of China; 4 Department of Otolaryngology, the Second Affiliated Hospital of Zhejiang University School of Medicine, Hangzhou, Zhejiang Province, People’s Republic of China; Stony Brook University, UNITED STATES

## Abstract

Cancer-associated fibroblasts (CAFs) play a crucial role in cancer progression and even initiation. However, the origins of CAFs in various cancer types remain controversial, and one of the important hypothesized origins is through epithelial-mesenchymal transition (EMT) from cancer cells. In this study, we investigated whether the HEp-2 laryngeal cancer cells are able to generate CAFs via EMT during tumor formation, which is now still unknown. The laryngeal xenografted tumor model was established by inoculating the HEp-2 laryngeal cancer cell line in nude mice. Primary cultured CAFs from the tumor nodules and matched normal fibroblasts (NFs) from the adjacent connective tissues were subcultured, purified, and verified by immunofluorescence. Migration, invasion, and proliferation potentials were compared between the CAFs and NFs. A co-culture of CAFs with HEp-2 cells and a co-injection of CAFs with HEp-2 cells in nude mice were performed to examine the cancer-promoting potential of CAFs to further verify their identity. Karyotypic analyses of the CAFs, NFs, and HEp-2 cells were conducted. A co-culture of NFs with HEp-2 cells was also performed to examine the expression of activated markers of CAFs. A pathological examination confirmed that the laryngeal xenografted tumor model was successfully established, containing abundant CAFs. Immunocytochemical staining verified the purities and identities of the CAFs and NFs. Although the CAFs manifested higher migration, invasion, proliferation, and cancer-promoting capacities compared with the NFs, an analysis of chromosomes revealed that both the CAFs and NFs showed typical normal mouse karyotypes. In addition, the NFs co-cultured with HEp-2 cells did not show induced expressions of activated markers of CAFs. Our findings reveal that the CAFs in the HEp-2 established laryngeal xenografted tumor are not of laryngeal cancer origin but of mouse origin, indicating that the HEp-2 laryngeal cancer cells cannot generate their own CAFs via EMT in this model.

## Introduction

The progression, metastasis, and even initiation of cancer are no longer recognized as independent events that are solely caused by genetic mutations and the uncontrollable growth of malignant cancer cells. The microenvironment of the local host tissue, which contains various types of stromal cells, has been recognized as an essential participant [1–3]. As the most abundant cell type in the tumor stroma, cancer-associated fibroblasts (CAFs) are recognized as playing a crucial role in cancer development by various mechanisms. They synthesize, degrade, and remold the extracellular matrix by secreting laminin and type IV collagen or proteases, such as matrix metalloproteinase; they secrete various soluble paracrine and autocrine growth factors that maintain the growth of tumor cells; and they mediate tumor-promoting inflammation [4–7]. In addition, CAFs have now been considered potential inducers in cancer initiation by providing oncogenic signals to the normal epithelia rather than acting as mere promoters in cancer progression [8].

Despite progress made in identifying the biological functions of CAFs in cancer development, there still exists a significant ambiguity with respect to their origins [4,9]. CAFs found in various cancers exhibit similar perpetually activated phenotypes, neither reverting back to a normal phenotype nor undergoing apoptosis [10]; however, they demonstrate a high degree of heterogeneity in their origins in different types of cancer [11]. They may be derived from cancer cells or normal epithelial cells through epithelial-mesenchymal transition (EMT), from the activation of resident normal fibroblasts (NFs) via genetic or epigenetic alteration induced by signals from adjacent tumor cells, from endothelial cells through endothelial to mesenchymal transition, or from bone marrow-derived hematopoietic stem cells or mesenchymal stem cells [4,12,13].

Among the possible origins, EMT from cancer cells is considered an important origin of CAFs [4,5,12]. By providing the proper conditions, breast cancer cells can transfer to myoepithelial cells and finally to myofibroblasts, the ancestors of CAFs [14]. By activating Ras and transforming growth factor-beta (TGF-β) signaling, the mouse squamous skin carcinoma cells can obtain mesenchymal morphology with the loss of adhesion marker E-cadherin [15]. Furthermore, Petersen et al. provide evidence that it is through EMT that breast cancer cells generate their own CAFs, which interact reciprocally with epithelial tumor cells to facilitate tumor growth [16].

Laryngeal cancer is one of the most common solid tumors of the head and neck region whose tumor stroma also contains abundant CAFs. We have previously isolated CAFs from primary cultured laryngeal cancerous tissue and demonstrated that the conditioned medium from CAFs promoted the proliferation, migration, and invasion of laryngeal cancer cells significantly [17]. However, whether the laryngeal cancer cells can generate their own CAFs via EMT remains unknown. In this study, we established a laryngeal xenografted tumor model in nude mice by using HEp-2 cells to mimic the process of tumor development. In addition, we investigated the differences in biological properties and karyotypes between primary cultured CAFs and NFs, and we concluded definitively that the HEp-2 laryngeal cancer cells could not generate their own CAFs via EMT during tumor formation in this model. Instead, the CAFs in this xenografted tumor are of mouse origin.

## Materials and Methods

### Ethics statement

The protocol on the care and use of laboratory animals was approved by the Shanghai Medical Experimental Animal Care Committee. All surgery was performed under sodium pentobarbital anesthesia, and all efforts were made to minimize suffering.

### Cell line and cell culture

The human laryngeal cancer cell line, HEp-2, was obtained from the Cell Bank of Type Culture Collection at the Chinese Academy of Sciences (Shanghai, China). The cells were maintained in Dulbecco's Modified Eagle's Medium (DMEM) (Invitrogen, Grand Island, NY, USA) supplemented with 10% fetal bovine serum (FBS) (Invitrogen) and 1% penicillin/streptomycin (Invitrogen) in a humidified 5% CO2 incubator at 37°C. The medium was renewed every three days.

### Establishment of laryngeal xenografted tumor model in nude mice

Six male BALB/c nude mice at six to eight weeks in age were purchased from the Slac Laboratory Animal Company (Shanghai, China). Cultured HEp-2 cells in the exponential phase were trypsinized by 0.25% trypsin-EDTA (Invitrogen) and suspended in phosphate buffered saline (PBS). Approximately 1×10^7^ HEp-2 cells suspended in 0.2 ml of PBS were injected into the subcutaneous space of the armpit of each mouse. The mice were fed in laminar flow cabinets under specific pathogen-free conditions and were palpated for tumor formation twice every week. Four weeks after injection, the mice were euthanized. All tumor nodules were resected and confirmed by hematoxylin and eosin (HE) staining.

### Primary culture and purification of CAFs from xenografted tumor and NFs from adjacent connective tissue

The resected tumor nodules and adjacent normal connective tissue were washed in triple antibiotic PBS containing 1% penicillin/streptomycin and amphotericin B (10 ug/ml) (Invitrogen) and cut into small fragments, followed by trypsinization in type IV collagenase (200 U/ml, Sigma, Saint Louis, MO, USA) overnight at 37°C. After washing in PBS, the digested fragments were suspended in DMEM supplemented with 15% FBS and 1% penicillin/streptomycin and seeded in 25-cm^2^ flasks (Corning Incorporated, Corning, NY, USA) in a humidified 5% CO2 incubator at 37°C. Two days later, the CAFs, NFs, and HEp-2 cells grew out of the fragments. The CAFs and NFs were purified by repeated brief exposure (within 5 min) to 0.25% trypsin-EDTA, a procedure termed differential trypsinization. Cells near confluence were trypsinized and subcultured. The matched NFs served as a control for the CAFs.

### Immunocytochemistry

Harvested CAFs and NFs were seeded onto glass coverslips, incubated for 24 h, rinsed in PBS, and then fixed with 4% paraformaldehyde for 15 min. This was followed by incubation in 5% bovine serum albumin containing 10% normal goat serum (Boster, Wuhan, China) (in the presence or absence of 0.3% Triton X-100 to permeabilize the cells) at room temperature for 40 min to block nonspecific interactions. The cells were then incubated with rabbit anti-mouse primary antibodies of pan-cytokeratin (CK) (1:200; Epitomics, Burlingame, CA, USA), vimentin (1:200; Epitomics), α-smooth muscle actin (α-SMA) (1:200; Epitomics), and fibroblast activation protein (Fap) (1:250; Abcam, Cambridge, UK) at 4°C overnight. After being washed, the cells were incubated with secondary antibodies (Jackson ImmunoResearch, West Grove, PA, USA) of fluorescein isothiocyanate (FITC)-conjugated goat anti-rabbit IgG (H+L) (1:100) or Cy^TM^3-conjugated goat anti-rabbit IgG (1:200) in the dark for 1 h at 37°C. The nuclei were stained with 4', 6-diamidino-2-phenylindole (DAPI) (Boster). Ten randomly selected microscopic fields (×200) of vimentin staining were analyzed to calculate the purities of the CAFs and NFs. Epithelial cells from the skins of the nude mice were primary cultured and served as positive control in pan-CK staining.

### Scratch wound healing assay of CAFs and NFs

The CAFs and NFs were seeded in a 24-well plate. The cell monolayer near confluence was gently scratched by a 10-μl pipette tip across the center of the well. While scratching, the long axial of the tip was kept perpendicular to the bottom of the well to create an equal gap distance. Another straight line, perpendicular to the first wound line, was scratched to create a cross. After scratching, each well was washed with PBS to remove the detached cells and replenished with fresh medium. Photos were taken, and a phase-contrast microscope was used at intervals of 0, 24, and 48 h to quantitatively measure the distances between the cell margins after scratching. To assess the exact speed of cell migration, the web-based automated WimScratch Image Analysis was used to measure the change in the cell-covered area over time, which was the characteristic parameter in migration assays. (http://ibidi.com/applications/wound-healing-and-migration/data-acquisition-and-analysis/).

### Invasion assay of CAFs and NFs

The invasion assay was performed with 24-well transwell chambers (pore size, 8μm) (Corning Incorporated, Corning, NY, USA) coated with Matrigel (BD Biosciences, Bedford, MA, USA). The lower compartment was added with DMEM (600 μl) supplemented with 15% FBS, and the upper chamber was inserted into the medium in the lower compartment. Approximately 2×10^4^ CAFs and NFs suspended in 200 μl of serum-free DMEM were plated in the upper chamber. The plates were then placed in the incubator at 37°C for 24 h before being taken out. The cells that did not migrate through the pores and remained on the upper side of the filter membrane were gently removed with a cotton swab. The cells that migrated through the Matrigel were fixed with methanol for 15 min, stained with 0.1% crystal violet for 15 min, rinsed with PBS, and counted under a microscope. Ten low-power fields (×100) were randomly selected for counting.

### Proliferation assay of CAFs and NFs

CAFs and NFs were cultured in a 96-well plate at a density of 3,000 cells/well in 0.2 ml of DMEM supplemented with 15% FBS and 1% penicillin/streptomycin for 6 d. The medium without cells served as a blank control. On days 0, 2, 4, and 6 after seeding, cell growth rate was measured using the cell counting kit-8 (CCK-8, Dojindo Laboratories, Kumamoto, Japan) according to the manufacturer’s instructions. The incubation lasted 3 h. The ultraviolet absorbance was measured at a wavelength of 450 nm. In addition, CAFs of passages 5, 10, 15, 20, and 25 were used to investigate the differences in proliferation capacities during the in vitro culture system, using the same abovementioned method.

### Influence of co-cultured CAFs on the wound healing capacity of HEp-2 cells

The scratch wound healing assay of the HEp-2 cells near confluence was performed and measured, as described above, with 24-well transwell chambers (pore size, 0.4μm) (Corning Incorporated) without Matrigel. The HEp-2 cells were seeded in the lower chamber, and the CAFs/NFs (approximately 2×10^4^ cells) were seeded in the upper chamber. Both the HEp-2 cells and the CAFs/NFs were cultured in DMEM supplemented with 15% FBS for 72 h. The results were compared with the HEp-2 cells cultured alone in DMEM supplemented with 15% FBS in the lower chamber.

### Influence of co-cultured CAFs on invasion capacity of HEp-2 cells

The invasion assay was performed as described above with 24-well transwell chambers. Approximately 2×10^4^ HEp-2 cells suspended in 200 μl of serum-free DMEM were seeded in the upper chamber. The lower chambers were seeded with CAFs/NFs (approximately 1×10^5^ cells) suspended in DMEM supplemented with 15% FBS or added with DMEM supplemented with 15% FBS. The HEp-2 cells cultured alone in the upper chamber served as a control.

### Influence of co-cultured CAFs on proliferation capacity of HEp-2 cells

The proliferation assay was performed by cell counting with 24-well transwell chambers (pore size, 0.4μm) (Corning Incorporated) without Matrigel. Approximately 1×10^4^ HEp-2 cells were seeded in the lower chamber, and the CAFs/NFs (approximately 2×10^4^ cells) were seeded in the upper chamber. Both the HEp-2 cells and the CAFs/NFs were cultured in DMEM supplemented with 15% FBS. On days 0, 2, 4, and 6 after seeding, the HEp-2 cells in the lower chamber were trypsinized for cell counting. The HEp-2 cells cultured alone in DMEM supplemented with 15% FBS in the lower chamber served as a control.

### Karyotype analysis

CAFs, NFs, and HEp-2 cells in the exponential growth phase were incubated with colchicine for 6 h at a concentration of 140 ng/ml, harvested by trypsinization, and suspended in 7 ml preheated hypotonic KCl solution (0.075 mol/l) for 30 min at 37°C. This was followed by the addition of 1 ml fresh fixing solution (methanol/acetic acid, 3:1, v/v) for 20 min. After centrifugation (1500 rpm×10 min), the cells were fixed twice in 5 ml fresh fixing solution for 30 min, dropped on cold glass slides, and stained with 10% Giemsa for 15 min. Then the slides were heated overnight at 60°C, put in 7.5% H_2_O_2_ for 3 min, treated by 0.05% trypsin, and stained with Leishman’s stain for 3 min. One hundred consecutive metaphases of each cell type were fully analyzed.

### Seven-day co-culture of NFs with HEp-2 cells

The co-culture was performed with 24-well transwell chambers (pore size, 0.4μm) (Corning Incorporated) without Matrigel. The HEp-2 cells were seeded in the upper chamber, and the NFs were seeded in the lower chamber. These two types of cells shared the same medium, which included DMEM supplemented with 15% FBS. At intervals on 1, 3, 5, and 7 days after seeding, the NFs in the lower chamber were trypsinized for the subsequent assays.

### Quantitative real-time polymerase chain reaction (qRT-PCR)

Total RNA was extracted from the NFs in the lower chamber of the co-culture system using the TRIzol^®^ reagent (Invitrogen). The PrimeScript^®^ RT reagent kit with the gDNA Eraser (Takara, Dalian, China) was used to perform the reverse transcription, and the SYBR^®^ Premix Ex TaqTM kit (Takara) was used to perform the qRT-PCR in a Lightcycler 480 instrument (Roche Diagnostics, Rotkreuz, Switzerland). The thermal cycling condition was as follows: an initial denaturation at 95°C for 30 s followed by 40 cycles at 95°C for 5 s and 60°C for 30 s. NFs cultured without HEp-2 cells and CAFs served as controls. Beta-actin (Actb) served as an endogenous control. The relative mRNA expression was calculated using the formula 2^-ΔΔCT^. The primers used are listed in Table 1.

**Table 1:** Primer sequences used for qRT-PCR.

Gene	Primer Sequence (5' → 3')	Gene ID	Accession No.	Product
α-SMA	(F) TAGAACACGGCATCATCA	11475	NM_007392	256 bp
	(R) CCAGAGTCCAGCACAATA			
Fap	(F) GAATGTCTCAGTCCTGTCTA	14089	NM_007986	183 bp
	(R) TAACCATCCTTGTCGCTAA			
Actb	(F) TTCCAGCCTTCCTTCTTG	11461	NM_007393	182 bp
	(R) GGAGCCAGAGCAGTAATC			

qRT-PCR, quantitative real-time polymerase chain reaction; F, forward; R, reverse.

### Western blot

Total protein from the lysates of the HEp-2 co-cultured NFs was analyzed with electrophoresis using sodium dodecyl sulfate-polyacrylamide gel (Beyotime, Shanghai, China). It was electrotransferred to a polyvinylidene fluoride membrane (Millipore, Billerica, MA, USA) and probed overnight using primary antibodies (Epitomics, Burlingame, CA, USA) for α-SMA (1:2000; Epitomics), Fap (1:2500; Abcam), and Actb (1:5,000; HuaAn Biotech, Hangzhou, China). The samples were then incubated in a secondary antibody of horseradish peroxidase-conjugated goat anti-rabbit IgG (H+L) (1:10,000) (Jackson ImmunoResearch). BeyoECL Plus (Beyotime) was used to perform signal detection. NFs cultured without HEp-2 cells and CAFs served as controls.

### Tumor-promoting capacity of CAFs and NFs

Twelve male BALB/c nude mice at six to eight weeks were randomly divided into two groups with 6 mice in each group. Briefly, cultured HEp-2 cells, CAFs, and NFs in the exponential phase were harvested and suspended in PBS. Approximately 1×10^6^ CAFs (mixed with 2×10^6^ HEp-2 cells) suspended in 0.2 ml of PBS were injected into the right armpit of each mouse (n = 6), and 1×10^6^ NFs (mixed with 2×10^6^ HEp-2 cells) injected the same way served as a control. Mice were euthanized four weeks after injection. All tumor nodules were photographed, weighed, fixed in 10% formalin, embedded in paraffin, and sectioned for HE staining.

### Statistical analysis

Data were presented as the mean ± standard deviation (SD) of three individual assays, and data were analyzed using the GraphPad Prism software version 5.00 for Windows (GraphPad Software, San Diego, CA, USA). The student’s t-test was used to perform statistical analysis and values of p less than 0.05 were considered statistically significant.

## Results

### Verification of laryngeal xenografted tumors by pathology

**Fig 1:**
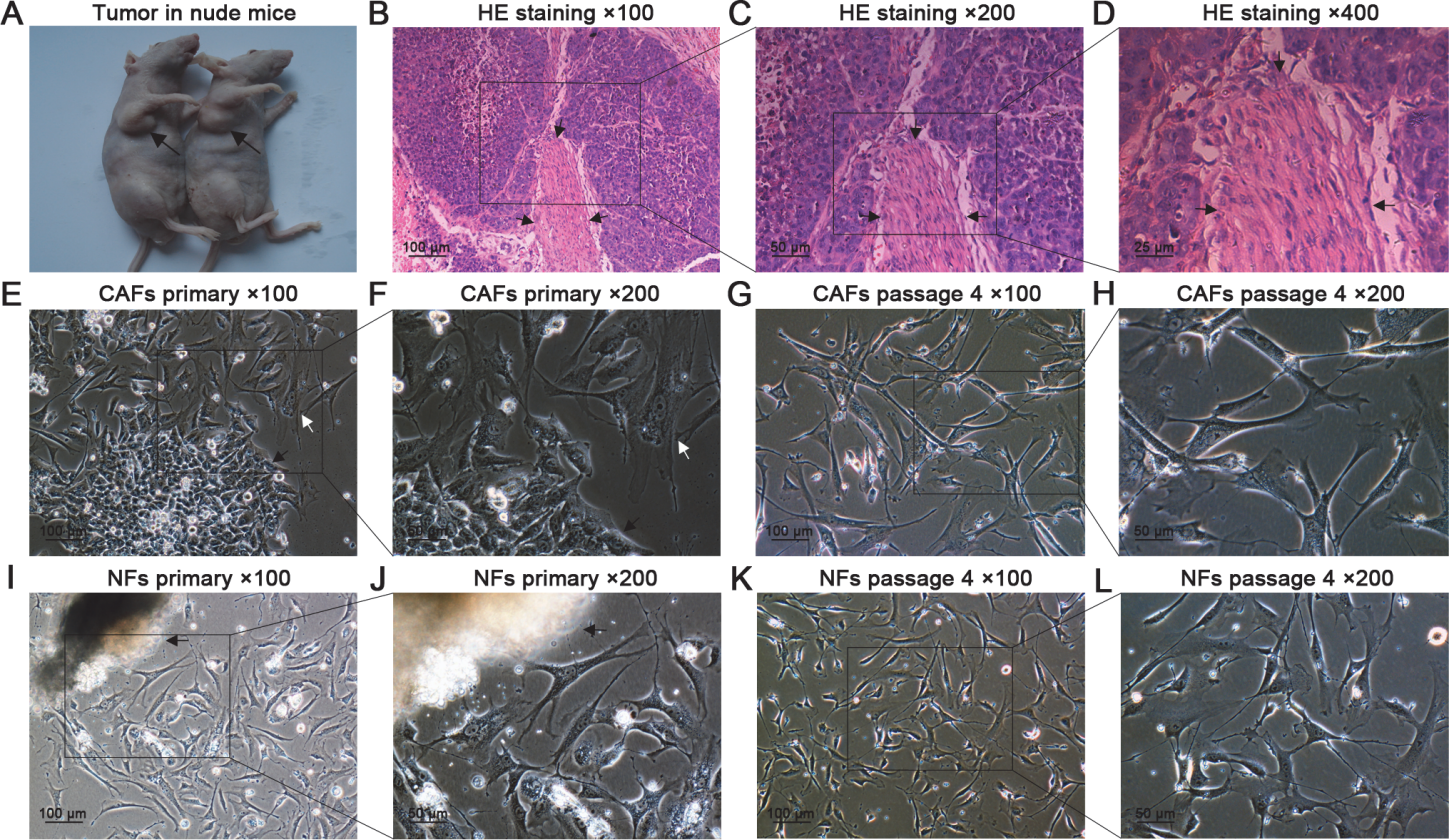
Tumor formation in nude mice, HE staining, and primary culture of CAFs and NFs. (A) Four weeks after injection, tumor nodules formed in the right armpits of nude mice (black arrows). (B–D) HE staining confirmed that the nodules were typical laryngeal squamous cell carcinoma containing abundant CAFs (black arrows). (E,F) Two days after seeding, the CAFs (white arrow) and HEp-2 cells (black arrow) grew out of the tissue fragments. (G,H) At passage 4, purified CAFs were obtained. (I,J) The NFs grew out of the fragment. (K,L) At passage 4, purified NFs were obtained.

Tumor nodules formed in the armpits (Fig. 1A) were resected and examined by HE staining (Fig. 1B–D), and the histological features were consistent with typical laryngeal squamous cell carcinoma, including numerous mitotic figures and a large nuclear to cytoplasmic ratio. The malignant epithelia were segregated and surrounded by extensive CAFs (indicated by arrows).

### Primary culture, subculture, and purification of CAFs and NFs

**Fig 2:**
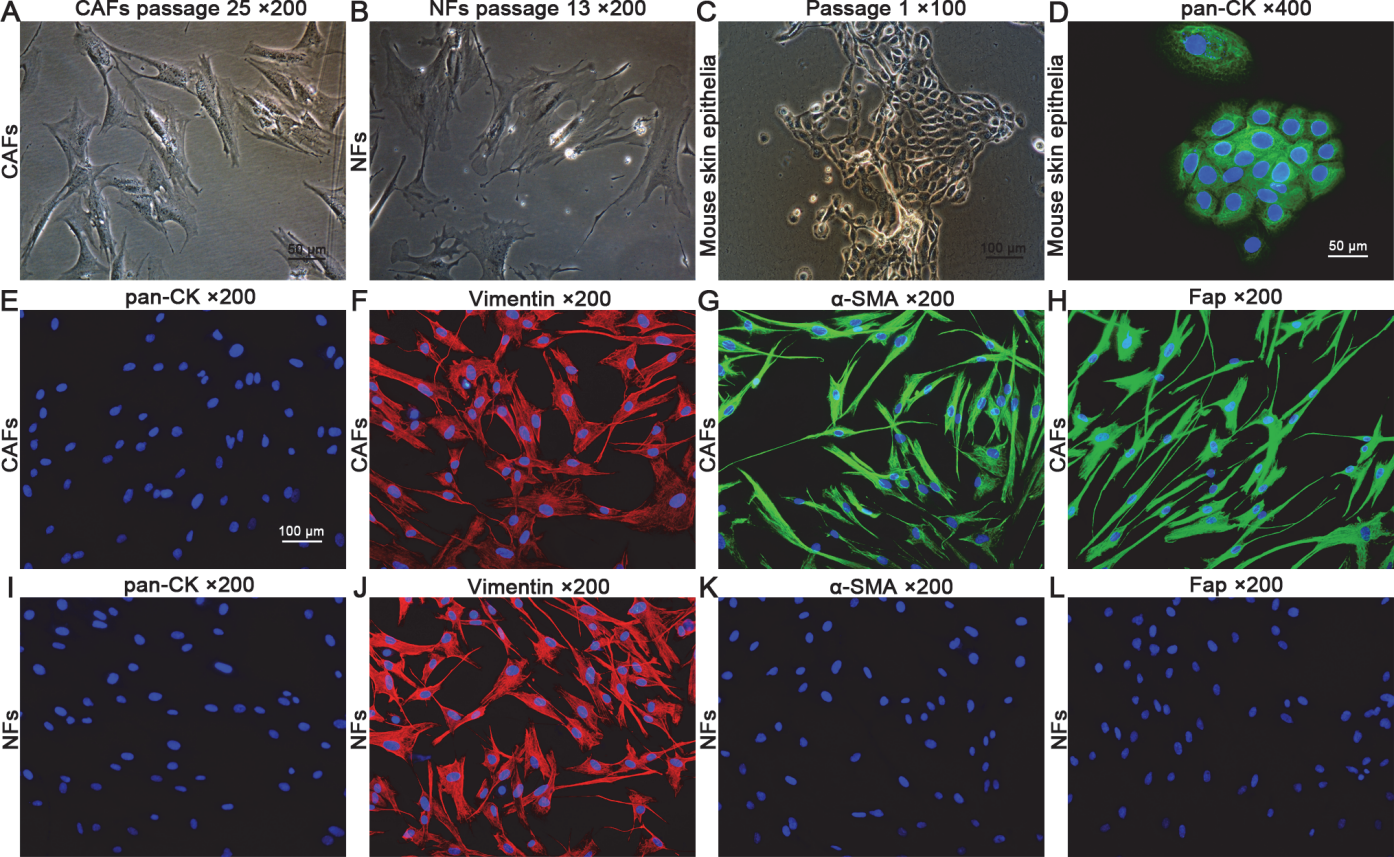
Cell morphology of CAFs, NFs, and mouse skin epithelia and immunocytochemical staining. (A,B) Cell morphology of CAFs (passage 25) and NFs (passage 13). The CAFs did not show significant morphological changes compared with the NFs before passage 25; however, the NFs showed changes in morphology, including an enlarged and more flattened outline, compared with the CAFs after passage 10. (C) Cell morphology of primary cultured mouse skin epithelia at passage 1. (D) The mouse skin epithelia showed positive staining of pan-CK (positive control). (E–L) The CAFs and NFs showed negative staining of pan-CK and positive staining of vimentin, indicating their fibroblast identities. Compared with the NFs, the CAFs showed positive staining of α-SMA and Fap, two markers of activated fibroblasts.

Two days after seeding, the CAFs, NFs, and HEp-2 cells grew out of the fragments. The CAFs and NFs were successfully purified by differential trypsinization within passages 3–4 and stored in liquid nitrogen at every passage, starting from passage 3. During subculture, the morphology of NFs did not show any changes before passage 10 (Fig. 1E–L); however, after passage 10, the morphology of NFs showed slight changes, including an enlarged and more flattened outline. In contrast, the CAFs did not show morphological changes before passage 25 (Fig. 2A, B). In addition, the NFs after passage 10 responded more slowly to trypsin during trypsinization than the CAFs did. Therefore, the subsequent assays were performed using CAFs and NFs within passage 10, unless otherwise specified.

### Purity and identity of CAFs and NFs verified by immunofluorescence

Both the CAFs and NFs showed negative staining of pan-CK and positive staining of vimentin, indicating their fibroblast identities. An analysis of 10 randomly selected microscopic fields (×200) of vimentin staining revealed that both the purity of CAFs and NFs was 100%. In addition, the CAFs also showed positive staining of α-SMA and Fap, two markers found in activated fibroblasts, compared with the NFs (Fig. 2C–L).

### Higher migration capability of CAFs

**Fig 3:**
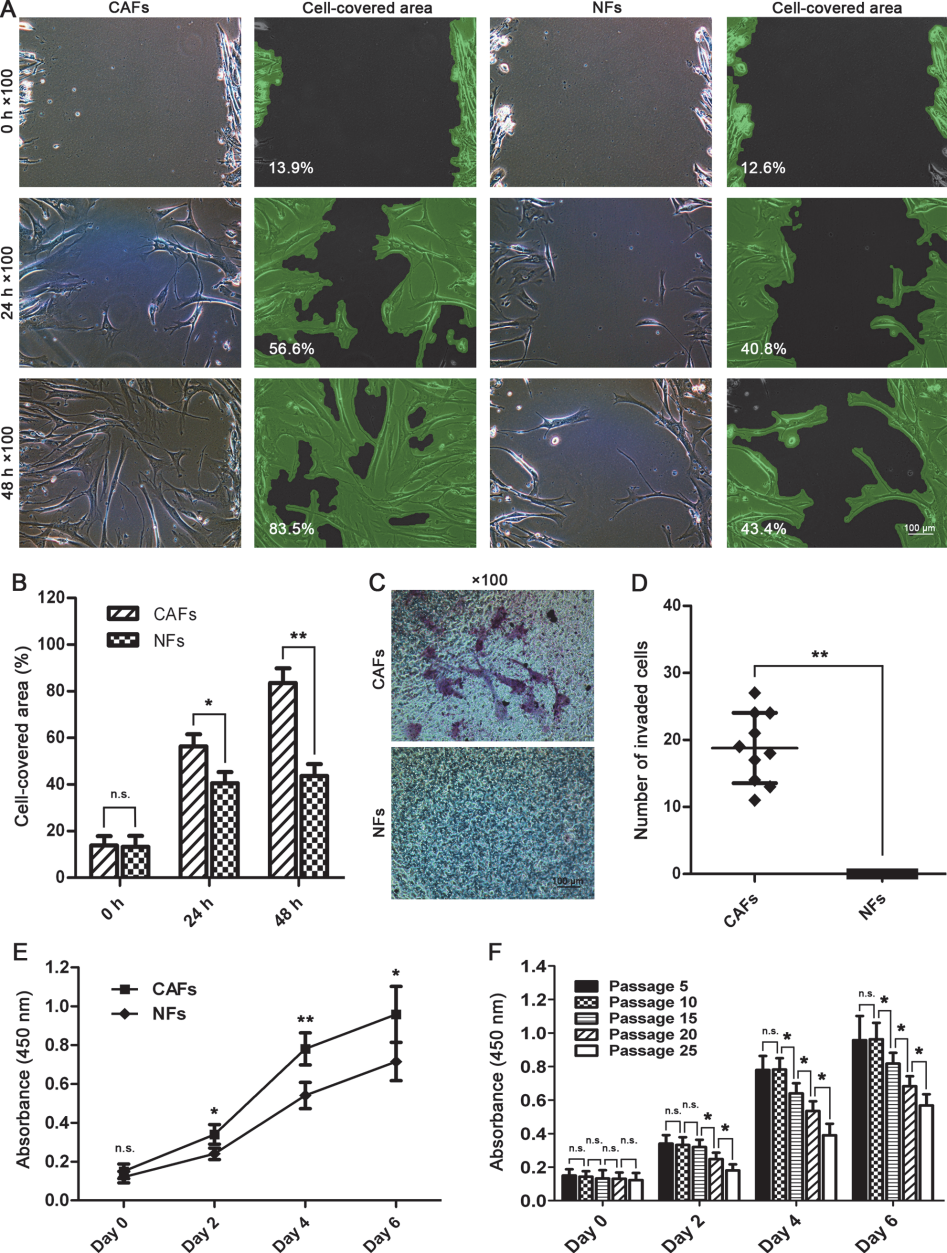
Isolated CAFs showed higher migration, invasion, and proliferation potentials than the NFs. (A,B) Gap closure assay and statistical analysis. The CAFs showed higher migration capacity compared with NFs and completely closed the gaps within 48 h. (C,D) Invasion assay and statistical analysis. The CAFs were found to be able to invade across the Matrigel; however, no NFs were found to invade across the Matrigel, indicating activated invasion potential of CAFs. (E) Cell growth rate measured by CCK-8 assay revealed that the CAFs had higher proliferation capacity than the NFs did. (F) Growth rate difference of CAFs in different passages. The CAFs began to show increasingly decreased proliferation capacity from passage 15 (^n.s.^p>0.05, *p<0.05, **p<0.01).

The differences in migration capacities were measured using gap closure assay. At 34 h after scratching, the CAFs began to manifest preferential speeds in gap shortening (*p<0.05); At 48 h, the CAFs completely closed the gaps (**p<0.01), indicating their higher migration potential compared with the NFs (Fig. 3A,B).

### Higher invasion capacity of CAFs

The 24-well transwell chambers were used to assess the differences in invasion potentials between the CAFs and NFs. CAFs were found to be able to migrate across the Matrigel; in contrast, no NFs were found to migrate across the Matrigel, indicating a higher invasion potential of CAFs compared with the NFs (**p<0.01) (Fig. 3C,D).

### Cell growth rate of CAFs and NFs

Cell growth rate was measured using CCK-8 assay at 0, 2, 4, and 6 days after seeding. On day 0, no significant difference in absorbance at 450 nm, which was consistent with the viable cell number, between CAFs and NFs was detected (^n.s.^p>0.05). However, on day 2, the CAFs began to show significantly higher absorbencies than the NFs (*p<0.05), and the difference became increasingly significant on days 4 (**p<0.01) and 6 (*p<0.05), indicating higher proliferation capacities of the CAFs (Fig. 3E). In addition, the CAFs began to show increasingly decreased proliferation capacity from passage 15 (*p<0.05), indicating that the CAFs alone cannot remain stable in the long-term in vitro culture system (Fig. 3F).

### Higher wound healing capacity of HEp-2 cells induced by co-cultured CAFs

**Fig 4:**
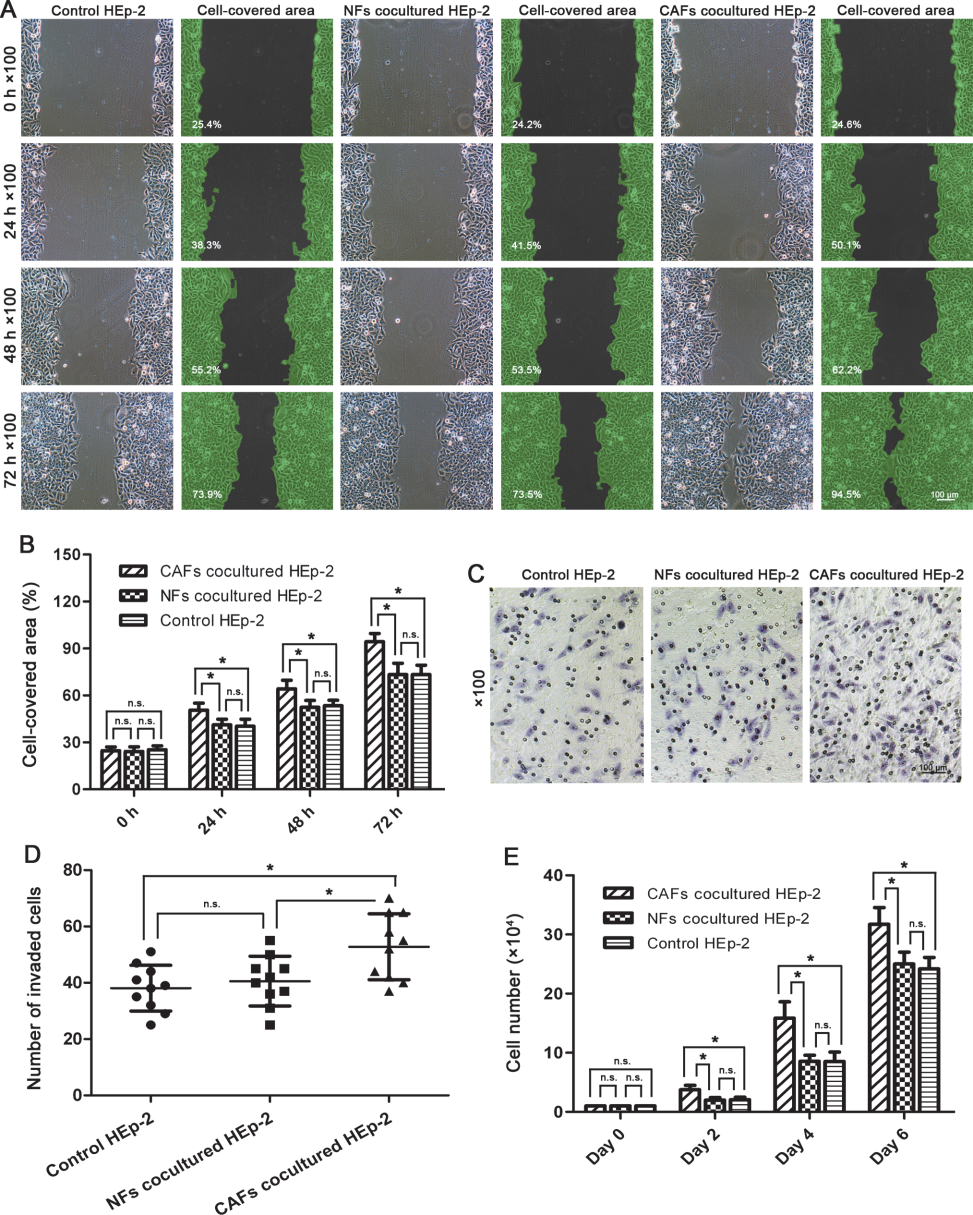
Co-cultured CAFs promoted the migration, invasion, and proliferation potentials of HEp-2 cells. (A,B) Wound healing assay and statistical analysis. The HEp-2 cells co-cultured with CAFs closed the gaps significantly faster compared with the control cells at time points of 24, 48, and 72 h. (C,D) Invasion assay and statistical analysis. The HEp-2 cells co-cultured with NFs had similar invasion capacity compared with those HEp-2 cells cultured alone. By contrast, the HEp-2 cells co-cultured with CAFs showed significantly higher invasion capacity than the control cells. (E) Cell growth rate measured by cell counting revealed that the co-cultured CAFs promoted the proliferation capacity of HEp-2 cells more than the NFs did (^n.s.^p>0.05, *p<0.05, **p<0.01).

The HEp-2 cells co-cultured with NFs showed similar migration capacity in the gap closure assay compared with those HEp-2 cells cultured alone (^n.s.^p>0.05). However, the gaps were closed significantly faster by the HEp-2 cells co-cultured with CAFs compared with the control cells at intervals of 24, 48, and 72 h (*p<0.05) (Fig. 4A,B), indicating that the migration potential of the HEp-2 cells was promoted by the co-cultured CAFs.

### Enhanced invasion capacity of HEp-2 cells induced by co-cultured CAFs

The HEp-2 cells co-cultured with NFs did not show significantly higher invasion capacity than HEp-2 cells cultured alone (^n.s.^p>0.05). By contrast, more HEp-2 cells co-cultured with CAFs were observed invading across the Matrigel compared with the control cells (*p<0.05) (Fig. 4 C,D), indicating that the co-cultured CAFs enhanced the invasion potential of the HEp-2 cells.

### Elevated proliferation capacity of HEp-2 cells induced by co-cultured CAFs

No significant differences in the cell numbers were detected between the HEp-2 cells co-cultured with NFs and the control HEp-2 cells on 0, 2, 4, and 6 days after seeding (^n.s.^p>0.05). However, the cell numbers of the HEp-2 cells co-cultured with CAFs increased much more significantly than the control cells on days 2, 4, and 6 (*p<0.05) (Fig. 4E), indicating elevated proliferation potential of the HEp-2 cells induced by the co-cultured CAFs.

### Chromosome analysis

**Fig 5:**
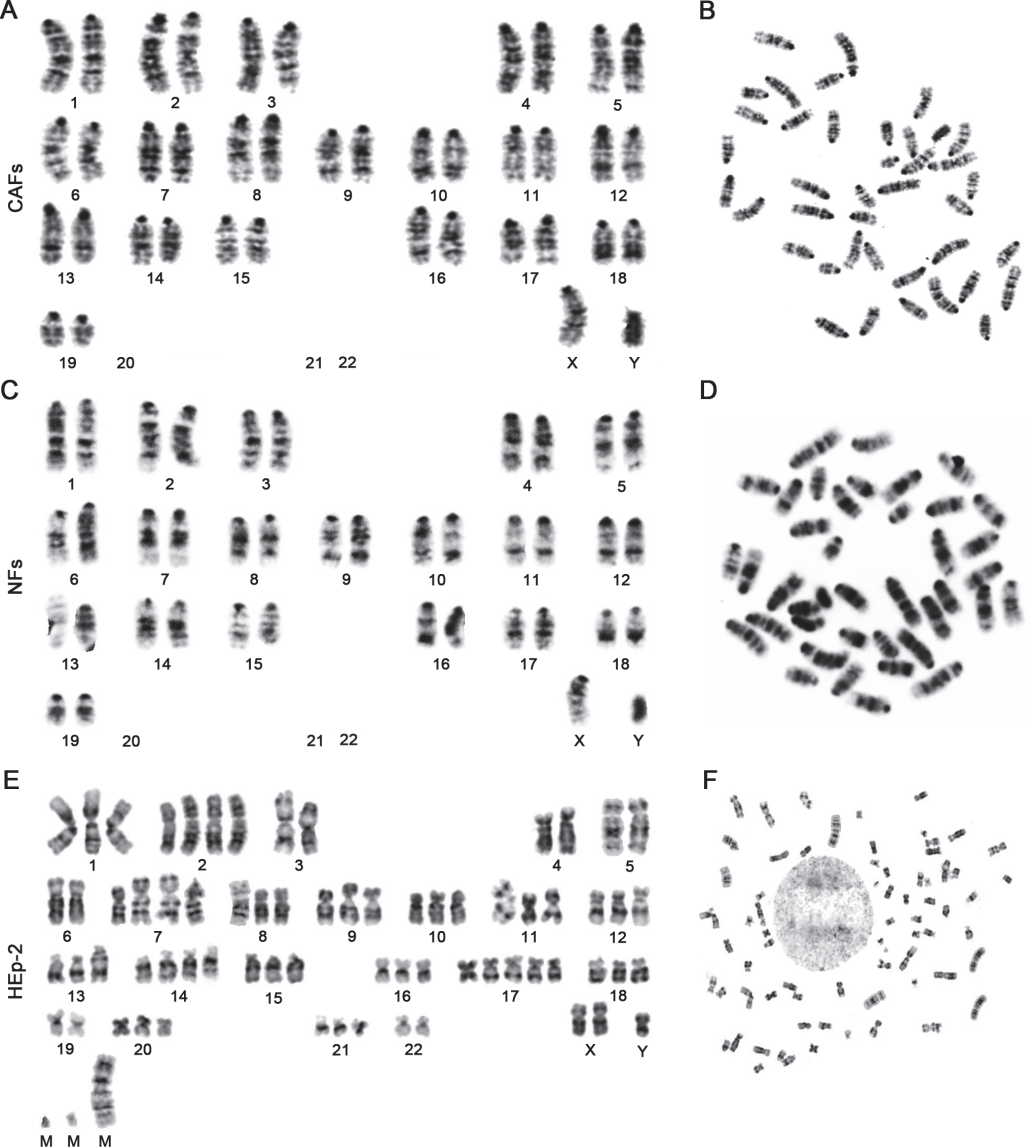
Representative karyotypes of CAFs, NFs, and HEp-2 cells. Both the CAFs (A,B) and NFs (C,D) showed typical characteristics of normal male mouse karyotype: acrocentric chromosome, modal number of 40 (38+XY), and without numerical or structural aberrations. (E,F) In contrast, the HEp-2 cell line showed a near-triploid karyotype, with both numerical and structural aberrations. Three marker chromosomes were consistently observed.

Chromosome analysis from 100 consecutive metaphases revealed that both the CAFs and NFs showed typical normal mouse karyotypes, without numerical or structural aberrations (Fig. 5A–D). By contrast, the karyotype of the HEp-2 cell line was near triploid, with both numerical and structural aberrations. Three marker chromosomes of unknown derivation were consistently observed (Fig. 5E,F), which was consistent with the previous reports [18,19] and served as a chromosome fingerprint to identify this cell line. These results indicated that both the CAFs and NFs were not derived from the HEp-2 cells, but they were of mouse origin. Representative karyotypes of CAFs, NFs, and HEp-2 cells are shown in Fig. 5.

### Expression of α-SMA and Fap in NFs after being co-cultured with HEp-2 cells

**Fig 6:**
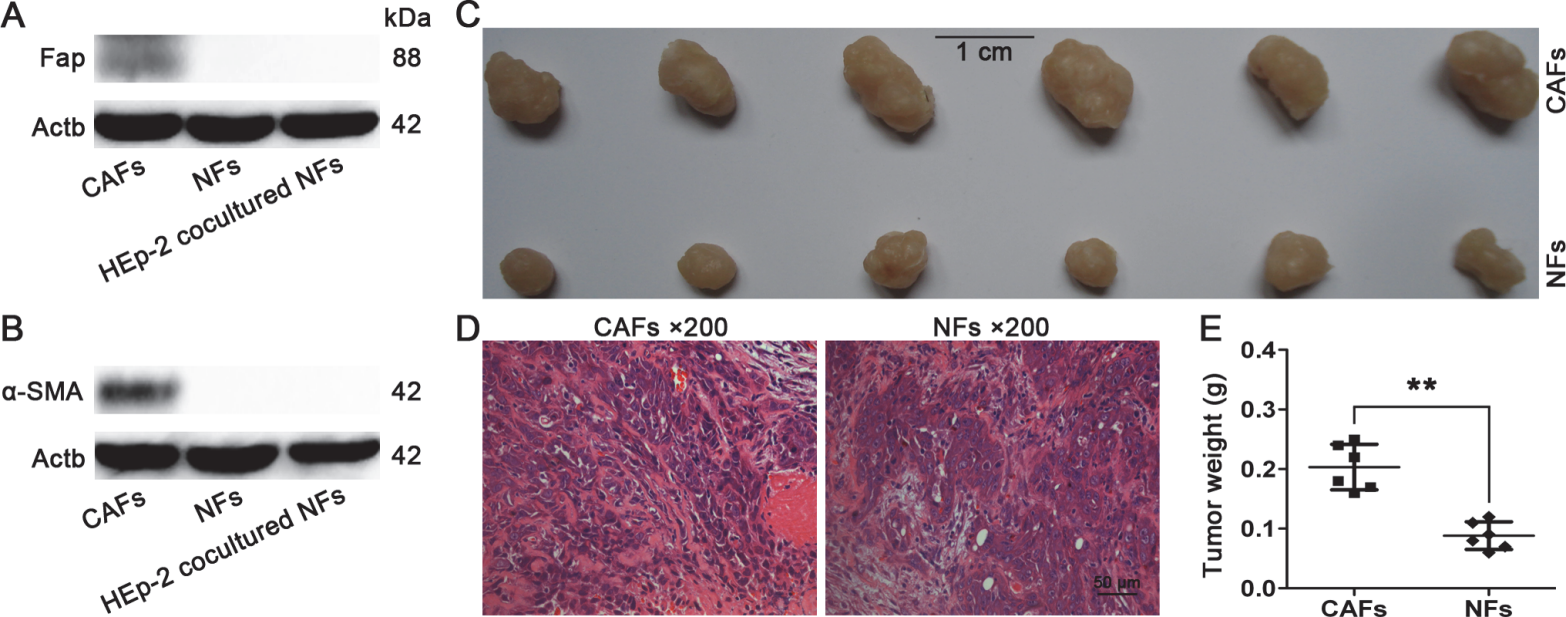
Western blot and tumor-promoting capacity of CAFs and NFs. (A,B) Protein levels of α-SMA and Fap in NFs after being co-cultured with HEp-2 cells for 7 d. The NFs and CAFs served as controls. The HEp-2 co-cultured NFs did not show elevated expressions of α-SMA and Fap. (C) Tumor nodules induced by CAFs and NFs (both mixed with HEp-2 cells) in nude mice. (D) Pathological examination revealed that all tumor nodules were typical laryngeal squamous cell carcinoma, and the tumor cells were surrounded by numerous CAFs. (E) Statistical analysis of tumor weights (**p<0.01).

After being co-cultured with HEp-2 cells for 1, 3, 5, and 7 d, the NFs did not manifest induced expressions of α-SMA and Fap in both mRNA (data not shown) and protein (Fig. 6A,B) levels, indicating that the NFs could not be transformed into CAFs in this co-culture system.

### Higher tumor-promoting capacity of CAFs

Both the CAFs and NFs mixed with HEp-2 cells successfully induced xenografted tumor nodules in the nude mice. All tumor nodules were confirmed by pathological examination. The CAFs mixed with HEp-2 cells induced larger tumor nodules compared with the NFs (**p<0.01), indicating that the CAFs harbored higher tumor-promoting capacity than did the NFs (Fig. 6C–E).

## Discussion

CAFs in the tumor microenvironment may be derived from various origins, and EMT from cancer cells is considered to be an important origin that accounts for a fraction of the CAFs that are present in tumors [4,5]. In this study, we investigated whether widely used laryngeal cancer cells, HEp-2, can generate their own CAFs in the xenografted tumor model via EMT. Because the karyotypic feature between human and mouse is significantly distinct, we may definitively answer this question by establishing a laryngeal xenografted tumor model in nude mice and comparing the karyotypic characterization of CAFs with that of NFs from the mice.

We successfully established this tumor model in nude mice and verified this model by pathological examination, which is consistent with a typical laryngeal squamous cell carcinoma. We also found that the xenografted tumors contained abundant CAFs, which segregated the malignant epithelial cells (Fig. 1B–D). To facilitate the following investigations, we cultured and purified the CAFs from the tumor nodules and the NFs from matched adjacent connective tissues (Fig. 1E–L). We found that purified CAFs propagated vigorously in vitro without significant changes in morphology during subculture before passages 25. In contrast, the NFs showed an increasingly enlarged and more flattened outline and increasingly reduced sensitivity to trypsin in trypsinization when they were subcultured for more than 10 passages (Fig. 2A,B), indicating that the NFs may preferentially undergo aging and apoptosis compared with the CAFs during long periods of subculture.

We subsequently verified the identities and purities of CAFs and NFs by immunofluorescence. Both the CAFs and NFs showed positive staining of vimentin and negative staining of pan-CK, indicating their definite identities of fibroblast.

Both the purities of CAFs and NFs analyzed by vimentin staining were 100%. It is reported that α-SMA and Fap are specific protein markers to identify CAFs [20]. We then immunochemically stained the CAFs with α-SMA and Fap antibodies and found that the CAFs showed positive staining of these two markers, indicating that they were verified CAFs (Fig. 2C–L).

It is reported that, compared with NFs, activated fibroblasts harbor enhanced capacity for migration due to elevated expressions of the α-SMA protein, increased capacity for invasion due to the secretion of active proteases to remodel the matrix, and increased capacity for proliferation [5,21]. We then compared the CAFs with the NFs for the differences in migration, invasion, and proliferation. We found that the isolated CAFs did harbor preferential capacity for migration, invasion, and proliferation (Fig. 3A–E), consistent with the above-mentioned reports, which further verified their identity as CAFs. Although the NFs showed migration capacity in the scratch wound healing assay (Fig. 3A,B), no NFs were found to migrate across the Matrigel compared with the CAFs (Fig. 3C,D) in our study, which may be due to the fact that the inactivated NFs could not produce any active proteases sufficient to degrade the Matrigel. For example, Fap functions as an active serine protease and plays a pivotal role in extracellular matrix remolding. However, expression of Fap is highly restricted to CAFs and activated stromal fibroblasts. It is not expressed in inactivated fibroblasts in normal tissue such as the NFs [22,23]. In our study, the NFs were also found to fail in the expression of Fap protein in the immunocytochemical staining assay (Fig. 2L) and the western blot assay of seven-day co-culture with the HEp-2 cells (Fig. 6A).

In addition, in order to investigate whether the CAFs would really remain biologically stable in the long periods of an in vitro culture system, CAFs of passages 5, 10, 15, 20, and 25 were used to detect the differences in proliferation capacity using the CCK-8 assay. We found that the CAFs began to show increasingly decreased proliferation capacity from passage 15 (*p<0.05), indicating that, in the absence of growth signals from the in vivo tumor microenvironment, it is impossible for the CAFs to remain biologically stable in the long periods of an in vitro culture system (Fig. 3F).

The CAFs are able to support the growth, invasion, and metastasis of cancer cells by secreting various cancer-promoting cytokines and growth factors into adjacent cancer cells [24–28]. Therefore, we further explored whether the isolated CAFs, compared with the NFs, had these biological functions by using the in vitro co-culture system. We found that a co-culture of CAFs with HEp-2 cells could significantly promote the migration, invasion, and proliferation potentials of HEp-2 cells (*p<0.05) (Fig. 4). In addition, we further investigated the differences in tumor-promoting activities between CAFs and NFs by injecting the CAFs and NFs (both mixed with HEp-2 cells) into the armpits of nude mice. We found that the CAFs mixed with HEp-2 cells induced larger tumor nodules compared with the NFs (**p<0.01), indicating that the CAFs had higher tumor-promoting activity than the NFs (Fig. 6C–E). These findings further verified their identity as CAFs.

In order to investigate whether the isolated CAFs in the laryngeal xenografted tumors are derived or partly derived from the HEp-2 cells via EMT, we next examined the karyotypes of the CAFs, NFs, and HEp-2 cells and found that both CAFs and NFs showed typical normal mouse diploid karyotype without numerical or structural aberrations (Fig. 5A–D). By contrast, the karyotype of the HEp-2cell line was near triploid, with three marker chromosomes consistently observed (Fig. 5E,F), which was consistent with the previous reports [18,19] and served as a chromosome fingerprint to identify this cell line. These results definitively indicate that CAFs in this tumor model are not derived from the laryngeal cancer cells via EMT.

It is reported that fibroblast activation during tumorigenesis involves a dynamic crosstalk between the NFs and other cells in the tumor microenvironment. The NFs are activated through direct contact with infiltrated immune cells, such as tumor-associated macrophages, and response to growth factors and cytokines secreted directly from the tumor cells, including fibroblast growth factor 2, platelet-derived growth factor, epidermal growth factor, and TGF-β [21,29]. The signals from the tumor cells play a crucial role in the activation of resident NFs into CAFs, which is another important source of CAFs. Furthermore, the NFs may be transformed into CAFs via co-cultivation with cancer cells for periods [4,9].

In this study, since CAFs in the laryngeal xenografted tumor model are not derived from laryngeal cancer cells via EMT, we next investigated whether the co-culture of NFs with laryngeal cancer cells could induce the expression of α-SMA and Fap, two activated markers of CAFs. Inconsistent with the previous reports, we did not find induced expression of these two markers in the co-cultured NFs in both mRNA (data not shown) and protein (Fig. 6A,B) levels, indicating that the co-culture of HEp-2 laryngeal cancer cells with NFs for 7 d could not transform the NFs into CAFs. The possible explanations may be: that the time course of 7 d is not long enough to induce the transformation; that some other cells in the tumor microenvironment and missing from the co-culture system, such as different types of immune cells, also play crucial roles in regulating the phenotype of CAFs; or that some other origins of the CAFs are not recapitulated by the co-culture model.

In conclusion, as the most abundant cell type in the cancer stroma and its abilities to promote and even initiate tumors, there is an increasing interest in studying CAFs as therapeutic targets. Although much remains to be clarified, our findings firmly consolidated the concept that the CAFs in the HEp-2 established laryngeal xenografted tumor are not derived from laryngeal cancer cells via EMT, although EMT from cancer cells is now recognized as an important hypothesized origin of CAFs in various cancer types, including breast cancer [14,16] and squamous skin carcinoma [15]. Further studies are urgently needed to clarify the origins of CAFs and the exact mechanisms by which NFs are activated, and thus CAFs are generated, in laryngeal cancer research.
