# Correction: Cancer-Associated Fibroblasts in a Human HEp-2 Established Laryngeal Xenografted Tumor Are Not Derived from Cancer Cells through Epithelial-Mesenchymal Transition, Phenotypically Activated but Karyotypically Normal

**DOI:** 10.1371/journal.pone.0128057

**Published:** 2015-05-04

**Authors:** Mei Wang, Chun-Ping Wu, Jun-Yan Pan, Wen-Wei Zheng, Xiao-Juan Cao, Guo-Kang Fan

The affiliations for the authors Jun-Yan Pan, Wen-Wei Zheng and Xiao-Juan Cao are incorrect. Jun-Yan Pan, Wen-Wei Zheng, Xiao-Juan Cao are not affiliated with #1 but with #2 Department of Otolaryngology, the Second Affiliated Hospital of Jiaxing University, Jiaxing, Zhejiang Province, People’s Republic of China.

## References

[1] Wang M, Wu C-P, Pan J-Y, Zheng W-W, Cao X-J, Fan G-K (2015) Cancer-Associated Fibroblasts in a Human HEp-2 Established Laryngeal Xenografted Tumor Are Not Derived from Cancer Cells through Epithelial-Mesenchymal Transition, Phenotypically Activated but Karyotypically Normal. PLoS ONE 10(2): e0117405 doi: 10.1371/journal.pone.0117405 25658113 10.1371/journal.pone.0117405 PMC4319834

